# Excessive unbound cefazolin concentrations in critically ill patients receiving veno-arterial extracorporeal membrane oxygenation (vaECMO): an observational study

**DOI:** 10.1038/s41598-021-96654-4

**Published:** 2021-08-20

**Authors:** Hendrik Booke, Otto R. Frey, Anka C. Röhr, Ute Chiriac, Kai Zacharowski, Tomas Holubec, Elisabeth H. Adam

**Affiliations:** 1 Department of Anesthesiology, Intensive Care Medicine and Pain Therapy, University Hospital Frankfurt, Goethe-University Frankfurt/Main, Theodor-Stern Kai 7, 60590 Frankfurt/Main, Germany; 2 Department of Pharmacy, Heidenheim General Hospital, Schloßhausstr. 100, 89522 Heidenheim, Germany; 3 Department of Pharmacy, University Hospital Heidelberg, Universität Heidelberg, Im Neuenheimer Feld 670, 69120 Heidelberg, Germany; 4 Department of Cardiac Surgery, University Hospital Frankfurt, Goethe-University Frankfurt/Main, Theodor-Stern Kai 7, 60590 Frankfurt/Main, Germany

**Keywords:** Continuous renal replacement therapy, Infection, Heart failure, Bacterial infection, Respiratory distress syndrome, Adverse effects, Drug therapy, Pharmacology, Clinical pharmacology, Pharmacokinetics

## Abstract

The scope of extracorporeal membrane oxygenation (ECMO) is expanding, nevertheless, pharmacokinetics in patients receiving cardiorespiratory support are fairly unknown leading to unpredictable drug concentrations. Currently, there are no clear guidelines for antibiotic dosing during ECMO. This study aims to evaluate the pharmacokinetics (PK) of cefazolin in patients undergoing ECMO treatment. Total and unbound plasma cefazolin concentration of critically ill patients on veno-arterial ECMO were determined. Observed PK was compared to dose recommendations calculated by an online available, free dosing software. Concentration of cefazolin varied broadly despite same dosage in all patients. The mean total and unbound plasma concentration were high showing significantly (*p* = 5.8913 E−09) greater unbound fraction compared to a standard patient. Cefazolin clearance was significantly (*p* = 0.009) higher in patients with preserved renal function compared with CRRT. Based upon the calculated clearance, the use of dosing software would have led to lower but still sufficient concentrations of cefazolin in general. Our study shows that a “one size fits all” dosing regimen leads to excessive unbound cefazolin concentration in these patients. They exhibit high PK variability and decreased cefazolin clearance on ECMO appears to compensate for ECMO- and critical illness-related increases in volume of distribution.

## Introduction

Extracorporeal membrane oxygenation (ECMO) is a well-established device for cardiorespiratory support in the most critically ill patients, which evolved quickly over the past decades and is now routinely performed in high volume cardiac surgery centers^1^. Although, it is a highly invasive system only used in the most critically ill patients more than 50% of ECMO-survival in cardiac patients is reported with increasing tendency over the last years^2^. Nevertheless, ECMO treatment is mainly used as a bridge to transplant or other long-term devices and consequently, patients receiving cardiorespiratory support are highly dependent on efficient pharmacologic treatment of complications or underlying diseases, such as infections.

Antibiotic treatment is a challenge in all critically ill patients: effective antimicrobial treatment depends not only on the right choice of antibiotics (empiric or calculated based on resistograms), but also on sufficient concentrations at the site of infection. For a therapeutic effect of a beta-lactam, such as cefazolin, the time above the minimal inhibitory concentration (MIC) for the most common pathogens is a crucial factor. In order to be able to evaluate time above the MIC, unbound concentrations of the drug need to be considered as it is the unbound fraction of the drug that can exert an effect.

However, pathophysiological changes in volume of distribution, drug clearance and protein-binding can be significantly different in critically ill patients, especially in patients on ECMO, compared to what is observed in other patient groups^3–6^. It is unknown to what extent the pharmacokinetics of cefazolin in ECMO patients differ from those of non-ECMO patients, hence, there are no clear guidelines for antibiotic dosing during ECMO treatment. Three additional main aspects can be thought of that alter PK in ECMO patients:

1. Reduced clearance (due to systemic inflammation because of the extracorporeal circuit)
2. Sequestration (due to pharmaceuticals binding to tubing and/or oxygenator)
3. Increased volume of distribution (Vd) (due to extracorporeal circulation and systemic inflammation)

Additionally, the physicochemical properties (lipophilicity/protein binding) of the used substance determine the influence of ECMO on PK. For cefazolin (Log_P_[− 0.6] and 60–92%), drug sequestration seems likely due to high expected protein binding, but unlikely due to low lipophilicity^7,8^.

Moreover, many pharmacological effects can be monitored and adjusted in real time (e.g. catecholamines—blood pressure) while the efficacy of antibiotic treatment can only be interpreted through surrogate parameters such as C-reactive protein (CRP), procalcitonin (PCT) and interleukin-6 (IL-6). Therefore, inappropriate treatment might be recognized too late. The use of dosing nomograms or dosing software with respect to pathophysiological changes that occur in severe infections might be an option to ensure therapeutic drug exposure. This study aimed to evaluate the pharmacokinetics of cefazolin in patients on ECMO. The secondary objective was to determine whether a dosing software enables ECMO patients to achieve target cefazolin exposures.

## Materials and methods

### Study design

This was a prospective, observational study in critically ill patients receiving ECMO and cefazolin admitted between 10/2019 and 10/2020 to the intensive care unit (ICU) of a German university hospital. Exclusion criteria were age < 18, mass transfusion and pregnancy. The study was approved by the local ethics committee (registration number: 493/17) and performed according to the principles of the Declaration of Helsinki and informed consent for participation was obtained for all patients. All patients received 6000 mg cefazolin over 24 h. The three single doses of 2000 mg were administered every 8 h for a period of 8 h. This way continuous infusion (same perfusion rate per minute over 24 h) of 6000 mg cefazolin over 24 h was obtained, which is recommended for beta-lactams by international guidelines^9^.

### Study procedures

Patients received at least 24 h of cefazolin infusion before inclusion. This way we made sure that all patients reached steady state before the first probe was taken. Blood sampling occurred at steady state, daily on day one, two, three and day six after inclusion using the indwelling arterial catheter. Each probe was taken at 10 a.m. and the time between probes of each patient was 24 h. Samples were centrifuged for 4 min at 3000 rpm (rounds per minute) immediately after sample collection and stored at − 80 °C until assay. Patient data were obtained from the medical record (PDMS, Metavision 5.4, iMDsoft, Tel Aviv, Israel) including weight, height, admission diagnosis, ECMO-settings, ventilatory parameters, blood gases results, laboratory results (hemoglobin, leukocytes, IL-6, albumin, creatinine and bilirubin), fluid balance, dialysis setting, Simplified Acute Physiology Score (SAPS II)^10^, length of ICU-stay, ECMO-survival and hospital survival. Glomerular filtration rate (GFR) was estimated (eGFR) in patients without continuous renal replacement therapy (CRRT = continuous dialysis) using creatinine concentrations and CKD-Epi-formula^11^.

CRRT was implemented according to current guidelines if necessary^12^. In these patients, eGFR was assumed as 15 ml/h. The *Calculator to Approximate Drug-Dosing in Dialysis* (CADDy; www.thecaddy.de; Dr. Otto Frey, Klinikum Heidenheim)^3,13^ was used as dosing software to predict cefazolin dose in patients considering the glomerular filtration rate, and if applicable, dialysis settings.

### Patients and ECMO management

In all patients vaECMO was implemented due to heart failure or combined heart–lung-failure. Standard cannulation sites were V. femoralis, A. axillaris and A. femoralis. Six patients received V. femoralis/A. axillaris cannulation and one patient received V. femoralis/A. femoralis cannulation. All patients received mechanical ventilation and critical care therapy as put forth by Hoyler et al.^14^ Antimicrobial therapy was administered upon admission on the ICU in all patients as prophylactic strategy after cardiothoracic surgery. In case of persistent critical ill condition (i.e. vaECMO) this prophylaxis was extended. All patients received 6000 mg cefazolin over 24 h. The three single doses of 2000 mg were administered every 8 h for a period of 8 h. This way continuous infusion (same perfusion rate per minute over 24 h) of 6000 mg cefazolin over 24 h was obtained, which is recommended for beta-lactams by international guidelines^9^.

Maquet® PLS (Permanent Life Support) system was used with Maquet® Polymethylpenthen-fiber oxygenator (PMP-oxygenator), Maquet® ROTAFLOW centrifugal pump and Maquet® BIOLINE coating (Heparin and Polypeptide coating). A Maquet® heat exchanger was used to keep body temperature at 37 °C. 1000 ml electrolyte-solution were used for circuit priming.

### Bioanalytical methodology

Total and unbound cefazolin concentrations were determined by a validated high-performance liquid chromatography (HPLC) assay with ultraviolet detection at 300 nm. The HPLC method was in line with the Valistat 2.0 (ARVECON GmbH, Germany) validation criteria as required by the German Society of Toxicology and Forensic Chemistry (GTFCh) and used to analyse clinical samples from patients treated with cefazolin^15^. The analysis was performed in the laboratory of the pharmacy department of Heidenheim General Hospital.

**Figure 1:**
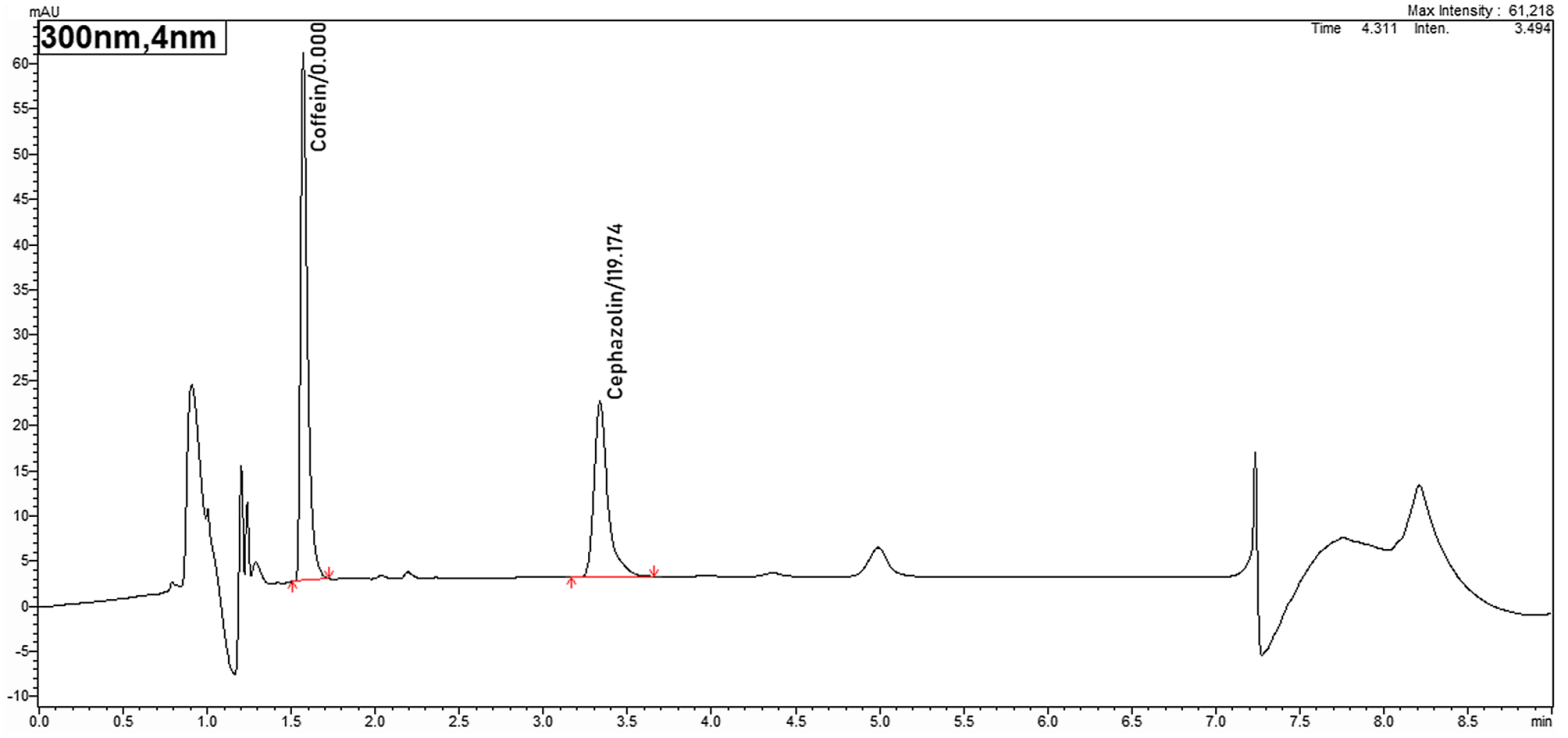
Chromatogram of total cefazolin concentration. This figure shows a typical HPLC (High Performance Liquid Chromatograph) chromatogram of cefazolin and the internal standard (caffein). The x-axis shows the run time in minutes while the y-axis demonstrates the absorption of the tested substance at 300 nm wavelength in absorbance units (AU). With a flow rate of 0.35 ml/min, cefazolin shows a retention time of 3.16 min in this setting.

In brief, the samples were thawed and centrifuged (4000 × *g*, 3 min) prior to ultrafiltration in order to remove possibly precipitated fibrin. 800 µl of plasma was pipetted into the sample reservoir of a Centrifree® tube (Ultrafiltration device with Ultracel® regenerated cellulose membrane, Merck KGaA, Germany). Then for 30 min the tube was centrifuged at 37 °C at 1000 × *g*. 100 µl plasma (or ultrafiltrate) were added to 500 µl of extraction-solvent (= Acetonitrile and Methanol 1:1 with 400 mg/L caffeine as internal standard) to precipitate proteins. This mixture was vortexted and then centrifuged at 8000 × *g* for 3 min. Afterwards, 100 µl of supernatant were diluted with 500 µl solvent A (0.1% formic acid in water HPLC grade). 10 µl of the solution was injected onto the HPLC–UV (Nexera-I 3D plus, Shimadzu; Germany). Chromatographic analysis was performed using a Shim-pack XR-ODS III; 2.2 µm; 150 × 2 column (Shimadzu, Germany) using an isocratic elution solvent A 77% and solvent B (0.1% formic acid in acetonitrile) 23% with a flow rate of 0.35 ml/min. In this setting, cefazolin has a retention time of 3.16 min (see Fig. 1). The assay was linear from 5.0 to 100 mg/L with a relative standard deviation (SD) for intra- and interday precision and accuracy < 10% at high, medium and low concentrations. The lower limit of quantification for cefazolin was 1 mg/L.

### Pharmacokinetic analysis

A therapeutic drug exposure was defined as unbound cefazolin concentration of 8–16 mg/L corresponding to four to eight times non-species related breakpoint of the European Committee on Antimicrobial Susceptibility Testing’s MIC90 data (http://www.eucast.org/ clinical_breakpoints: ECOFF cefazolin 2 mg/L)^16^. This exposure is recommended for beta-lactam antibiotics in order to achieve optimal clinical response^17^. A one-compartment model was used to perform PK analyses because cefazolin has a small volume of distribution (VD of ca. 0.2 L/kg), a protein binding of 60–92% and is essentially excreted by the kidneys (renal excretion 90%)^18^. Plasma concentrations in steady-state were the observed values. Observed cefazolin clearance (CL) was calculated using the following equation: $CL\left[ {{\text{L}} \cdot {\text{h}}^{ - 1} } \right] = \frac{{dose\left[ {{\text{mg}}} \right] }}{{24\,{\text{h}}}} \cdot c^{ - 1} \left[ {{\text{mg}} \cdot {\text{L}}^{ - 1} } \right]$. Predicted plasma concentration was calculated using following equitation: $c\left[ {{\text{mg}} \cdot {\text{L}}^{ - 1} } \right] = \frac{{dose\;CADDy \left[ {{\text{mg}}} \right] }}{{24\,{\text{h}}}} \cdot CL^{ - 1} \left[ {{\text{L}} \cdot {\text{h}}^{ - 1} } \right]$.

### Statistics

We used Microsoft® Excel® V16.35 for all our statistical analyses. Discrete variables are expressed as counts (percentage) and continuous variables as means ± standard deviation (SD). A Kolmogorov–Smirnov-test was used to verify the normality of distribution of our results. A two sample-t-test was used to compare clearance of CRRT-patients with clearance of patients not on CRRT. A one sample t-test was used to compare the amount of protein binding in comparison to the expected value according to the summary of product characteristics. A *p*-value of < 0.05 was considered as significant.

### Ethics approval and consent to participate

The study was approved by the local ethic committee (Ethikkommission des Fachbereichs Medizin der Goethe Universität Frankfurt) (registration number: 493/17). Consent for participation was obtained for all patients.

## Results

Patients receiving cefazolin and cardiorespiratory support (vaECMO) were mostly (n = 5) cardiac surgery patients with one patient needing vaECMO due to pulmonary fibrosis. Four patients needed CRRT during the study period. Only one patient absolved the full observational period of 6 days and four measurements, the other observations ended prematurely due to death, termination of ECMO-therapy or change in anti-infective therapy.

Therefore, we analyzed 17 test-results in total (11 with CRRT and 6 without CRRT).

Patient characteristics are shown in Table 1. Four out of six patients (66%) survived ECMO-therapy, with one patient dying under impella-therapy 5 days after ECMO-explantation.

**Table 1:** Patient and clinical characteristics.

Patients included	n = 5
Age, y	66 ± 6.6
Male sex	5 (83%)
Bodyweight, kg	91.0 ± 34.3
BMI	32.1 ± 10.6
SAPS II	54 ± 17
Patients on CRRT	4 (66%)
Dialytic flow, ml/h	2500 ± 341
Daily balance, ml	− 155 ± 1683
Albumin concentration, g/dl	2.1 ± 0.3
Cefazolin clearance, L/h	4.3 ± 3.9
Cefazolin clearance in patients with CRRT, L/h	2.0 ± 0.4
Cefazolin clearance in patients without CRRT, L/h	8.5 ± 3.8
Total cefazolin concentration, mg/L	98.1 ± 52.6
Free cefazolin concentration, mg/L	51.1 ± 33.8
Cefazolin free fraction, %	49 ± 9

Data are given as mean + / − s.d. or count and percentage as indicated. *Y* years, *kg* kilogram, *BMI* body mass index, *SAPS II* Simplified Acute Physiology Score II, *CRRT* continuous renal replacement therapy, *ml* milliliter, *h* hour, *g* gram, *dl* deciliter, *L* liter, % = percentage.

ECMO-treatment was practiced according to guidelines^19^ with a mean blood flow of 3.9 ± 1.3 L/min s.d. and medium gas flow of 2.9 ± 1.3 L/min s.d.. Oxygenation and decarboxylation through the ECMO enabled lung-protective ventilation with a target range for mean tidal volume (V_t_) of 4–6 mL/kg bodyweight. Fluid balance varied broadly with a mean daily balance of − 155 ± 1683 ml/d s.d.. Mean dialytic flow was ca. 2500 ml/h (± 341 ml/h s.d.).

Serum albumin was reduced in all of our patients (mean 2.1 g/dl ± 0.3 g/dl s.d.).

**Figure 2:**
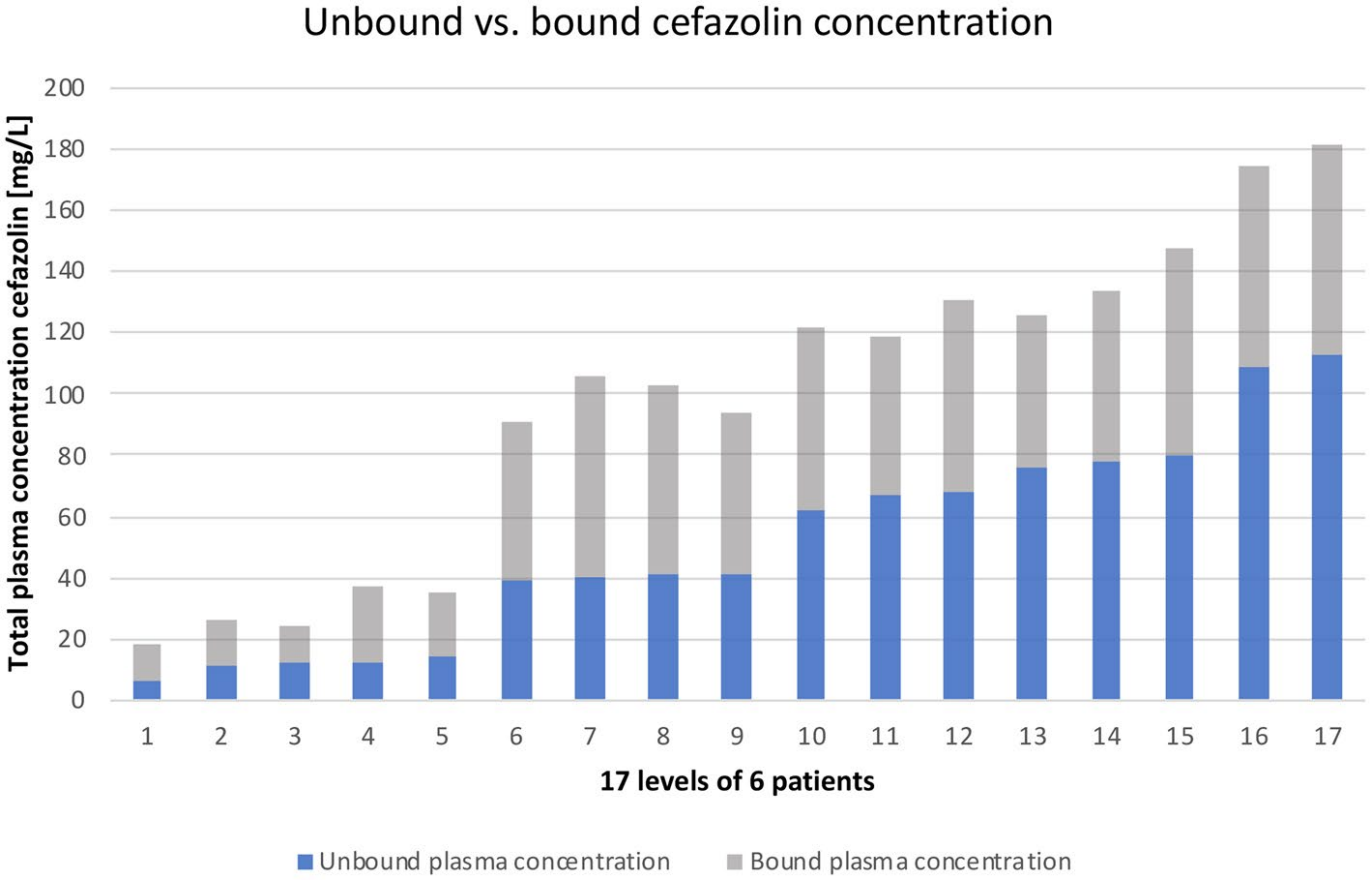
Unbound versus bound cefazolin concentration. This is a visualization of our measured plasma concentrations of cefazolin in ascending order of the unbound concentration. The y-axis shows the concentration of cefazolin in mg/L. Each bar represents one probe and is divided into the bound fraction (grey) and unbound fraction (blue) of the total cefazolin concentration. This diagram has two key messages: First, the unbound fraction is different in each probe and second, although some probes show high total concentration of cefazolin, the biologically active concentration (= unbound concentration in blue) is lower than in some probes with less total concentration (compare bar 10 and 11).

**Figure 3:**
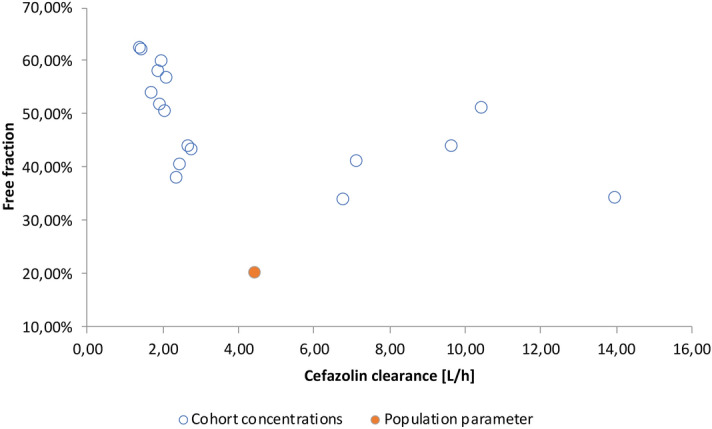
Cefazolin clearance and free fraction. This figure demonstrates the clearance in L/h (y-axis) and free fraction in % (x-axis) of cefazolin in comparison to population parameters. It shows the great variability of cefazolin clearance in our patients and that all measurements show greater unbound fraction than expected regarding the professional information of cefazolin.

**Figure 4:**
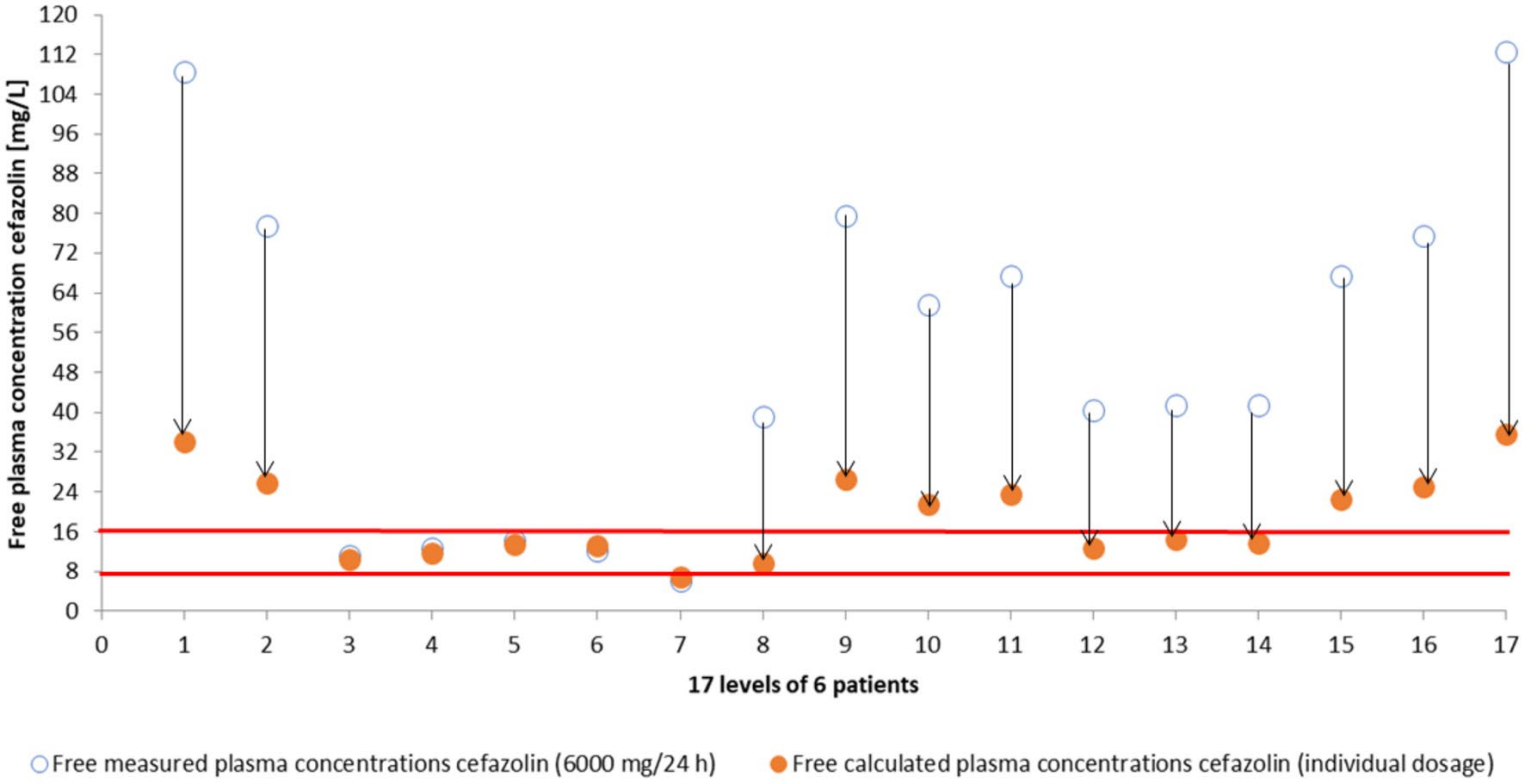
Effect of dosing software on unbound cefazolin concentration. By entering renal parameters and dialysis settings in a dosing software (thecaddy.de)^11^ we received a recommended dose of cefazolin for each patient and each day. We were then able to calculate the hypothetical plasma concentration using the recommended dose and the measured cefazolin clearance of that day. This figure shows the actual measured free plasma concentrations of cefazolin (blue circles) using 6000 mg/d and the calculated concentration if we had used dosing software (orange circles). The y-axis shows the free plasma concentration of cefazolin in mg/L and the red lines show the target range of (unbound) cefazolin with 4 times and 8 times ECOFF (2 mg/L).

Plasma concentrations and clearance of cefazolin showed great variety with a mean total and unbound plasma concentration of 98.1 mg/L ± 52.6 mg/L s.d. and 51.1 mg/L ± 33.8 mg/L s.d., respectively (see Fig. 2). The mean clearance was 4.3 L/h ± 3.9 L/h s.d.. The amount of unbound cefazolin in comparison to total cefazolin concentration altered as well (49% ± 9% s.d.) and was significantly (*p* = 5.8913 E−09) higher than the value from the corresponding summary of product characteristics (8–38%) (see Fig. 3) ^18^. All but one unbound plasma levels maintained above 4xECOFF (= 8 mg/l), 4 samples (24%) were in the main target range. 71% (n = 12) samples showed excessive exposure to unbound cefazolin > 8xECOFF (> 16 mg/L). Clearance of cefazolin was significantly (*p* = 0.009) higher in patients with preserved renal function (= not on CRRT) than in patients with CRRT (8.5 L/h ± 3.8 L/h s.d. vs. 2 L/h ± 0.4 L/h s.d.). Dose adjustment in consideration of renal function and RRT using CADDy software would have let to lower dosages but still sufficient concentrations of 18.9 mg/L ± 8.7 mg/L s.d. (see Fig. 4).

## Discussion

Our study shows high plasma concentrations of cefazolin with a considerable variability in critically ill patients receiving ECMO-treatment. Furthermore, we found higher unbound fractions compared to the summary of product characteristics^18^. As described elsewhere, critically ill patients show altered pharmacokinetics but it is still fairly unknown to what extend these are effected by ECMO-treatment^20–23^. That’s why there are no established treatment regimens for ECMO-patients and usually, dosing for none-ECMO patients is used instead.

Here, all patients received the recommended higher standard dose of cefazolin for gram-negative pathogens regardless of their renal function. This might be caused by the fear of underdosing antibiotics in these patients as further infection may lead to more complications (“the more, the better”). However, the benefit of free concentrations above 16 mg/L is debatable with increased risk of side effects^17^. As we stated before, it is extremely difficult to measure effects of sufficient anti-infective treatment leaving the clinician in the blind on whether the amount of administered antibiotic is sufficient or not.

Albumin concentrations in our patients were low which could explain the higher unbound fractions of cefazolin which we observed. Although low albumin concentrations are common in critically ill patients, further albumin sequestration and dilution due to the ECMO-circuit could be possible, as proteins bind to artificial surfaces such as ECMO-tubing and membrane oxygenators^24,25^. Regardless of modern coatings and PMP-oxygenators, ECMO-circuits still show accumulation of blood components over time which leads to *membrane fouling* ^26^. At the same time our findings endorse previous study-results that showed, predicting unbound fraction by using albumin concentration is probably not accurate as similar albumin concentrations in our patients still led to highly variable plasma concentrations^24,27,28^. That is why measuring the unbound fraction of cefazolin seems to be important as only then the biologically active concentration is determined. High total plasma concentrations with high protein binding, for example, might still lead to insufficient active (= unbound) cefazolin levels and would be unrecognized, if only the total concentration is measured. On the other hand, excessive cefazolin concentrations enhance the risk for adverse effects while the benefit of unbound concentrations above 16 mg/L is debatable^17^. Neurological symptoms (e.g. encephalitic signs, behavioral disorder and/or tonic clonic seizures) have been described in connection with high dosage of cephalosporins with cefazolin showing a lower neurotoxicity threshold than other beta-lactams^17,29^.

We also showed that dose adjustment for renal function and applied RRT (renal replacement therapy) using dosing software would be safe in this patient clientele. According to the free available used software (theCADDy.de^13^) cefazolin dosage could have been reduced in all but one patient while still maintaining sufficient plasma levels. This might even have the positive side effect of saving resources with cefazolin currently (February 2021) regarded as *“in shortage”* by the FDA (Food and Drug Administration) and ongoing supply difficulties for cephalosporins in other countries^30,31^. With routine therapeutic drug monitoring (TDM) the amount of needed antibiotic could have been reduced even further.

Finally, PK-studies showed that lipophilicity and protein binding are the main variables that determine ECMO-induced PK-changes^7,8^. Lipophilic and high protein binding drugs seem to show greater sequestration in ECMO-circuits. Although, there is no data for cefazolin (Log_P_[− 0.6] and 60–92%), drugs with similar lipophilicity and protein binding like vancomycin (Log_P_[− 3.1] and 55%) or ceftriaxone (Log_P_[− 1.7] and 85–95%) show concentration reductions of 8%, respectively 20%, in 24 h due to sequestration^31^. This might lead to further drug exposure after treatment termination which can then lead to prolonged interactions with other drugs and formation of resistance in pathogens because of insufficient antibiotic concentration. Reducing the excessive amount of cefazolin could mean lower sequestration and therefore less drug recovery from ECMO-circuit. However, one case report, which studied the pharmacokinetics of cefazolin, showed no reduction of cefazolin in post ECMO-membrane probes compared to pre-filter probes^32^.

All in all, our study shows that optimal dosing of cefazolin in patients with cardiorespiratory support is extremely difficult. While dosing software makes up for change in renal function, TDM-guided dosing might take this a step further and compensate for the great variability of pharmacokinetics in these most critically ill patients. Furthermore, the distinction between unbound and bound fractions of cefazolin offers a more precise measurement of active antibiotic and thus a better understanding of drug exposure in these patients. The here described method of measuring total and unbound cefazolin is easy, cost-efficient and fast, with results of free fraction cefazolin 1–2 h after blood sampling. This allows clinicians an almost real-time parameter for adequate cefazolin dosing in these critically ill patients.

Of course, this study has its limitations. Even though, ECMO-therapy is used more frequently, the number of patients remain rare. Thus, we were able to include only six patients into our study. Furthermore, not all patients completed the full observation period (e.g. due to change in antibiotic choice). This could be seen as a result of this highly unstable patient-clientele, which frequently requires change in therapy.

## Conclusion

Our study shows excessive unbound cefazolin concentrations in patients receiving extracorporeal cardiorespiratory support. Furthermore, we found a large variability in plasma concentration of cefazolin. As PK of cefazolin is mainly dependent on renal function and CRRT, the use of a dosing software helps the clinician in optimizing cefazolin dosing. Therapeutic drug monitoring could have been helpful for further dosing improvement and therefore minimizing the risk of potential side effects. When using TDM, the distinction between unbound and bound cefazolin is recommended as only the unbound fraction of cefazolin is biologically active.

## Data Availability

The dataset used and analyzed during the current study are available from the corresponding author on reasonable request.

## Abbreviations

BMI: Body mass index
CADDy: Calculator to Approximate Drug-Dosing in Dialysis
CL: Clearance
CRP: C-reactive protein
CRRT: Continuous renal replacement therapy
dl: Deciliter
ECMO: Extracorporeal membrane oxygenation
ECOFF: EUCAST epidemiological cut-off values
eGFR: Estimated glomerular filtration rate
EUCAST: European committee on antimicrobial susceptibility testing
FDA: Food and Drug Administration
g: Gram
GFR: Glomerular filtration rate
GTFCh: German Society of Toxicology and Forensic Chemistry
h: Hour
HPLC: High-performance liquid chromatography
ICU: Intensive care unit
IL-6: Interleukin-6
L: Liter
MIC: Minimal inhibitory concentration
ml: Milliliter
PCT: Procalcitonin
PK: Pharmacokinetics
PLS: Permanent life support
PMP-Oxygenator: Polymethylpenthen-fiber oxygenator
PTT: Prothrombin time
RASS: Richmond Agitation and Sedation Scale
rpm: Rounds per minute
RRT: Renal replacement therapy
SAPS II: Simplified Acute Physiology Score II
SD: Standard deviation
TDM: Therapeutic drug monitoring
vaECMO: Veno-arterial extracorporeal membrane oxygenation
Vd: Volume of distribution
V_t_: Tidal volume
Y: Year
